# 376. A Target Trial Emulation of Short vs Long Antibiotic Duration for the New Definition of Uncomplicated UTI

**DOI:** 10.1093/ofid/ofaf695.123

**Published:** 2026-01-11

**Authors:** Matthew Steinberger, Tejal N Gandhi, David Ratz, Tawny Czilok, Tara Pearlman, Jennifer Horowitz, Elizabeth McLaughlin, Ashwin Gupta, Steven J Bernstein, Anurag Malani, Danielle Osterholzer, Valerie M Vaughn, Scott A Flanders, Lindsay A Petty

**Affiliations:** University Of Michigan, East Lansing , MI; Michigan Medicine, Ann Arbor, Michigan; VA Ann Arbor Health System, Ann Arbor, Michigan; Michigan Medicine, Ann Arbor, Michigan; University of Michigan, South Lyon, Michigan; Michigan Medicine, Ann Arbor, Michigan; Michigan Medicine, Ann Arbor, Michigan; Michigan Medicine, Ann Arbor, Michigan; University of Michigan, South Lyon, Michigan; Trinity Health Michigan, Ann Arbor, Michigan; Hurley Medical Center/Michigan State University College of Human Medicine, Flint, Michigan; University of Utah, Salt Lake City, Utah; University of Michigan/Michigan Medicine, Ann Arbor, Michigan; University of Michigan, South Lyon, Michigan

## Abstract

**Background:**

IDSA complicated urinary tract infection (cUTI) guidelines posted for public comment now define cUTI as infection beyond the bladder. This change categorizes many patients previously considered cUTI as uncomplicated (uUTI), making more patients appropriate for short antibiotic treatment. We sought to assess differences in clinical outcomes for the short vs long treatment durations in patients meeting the new uUTI definition.

**Figure:**
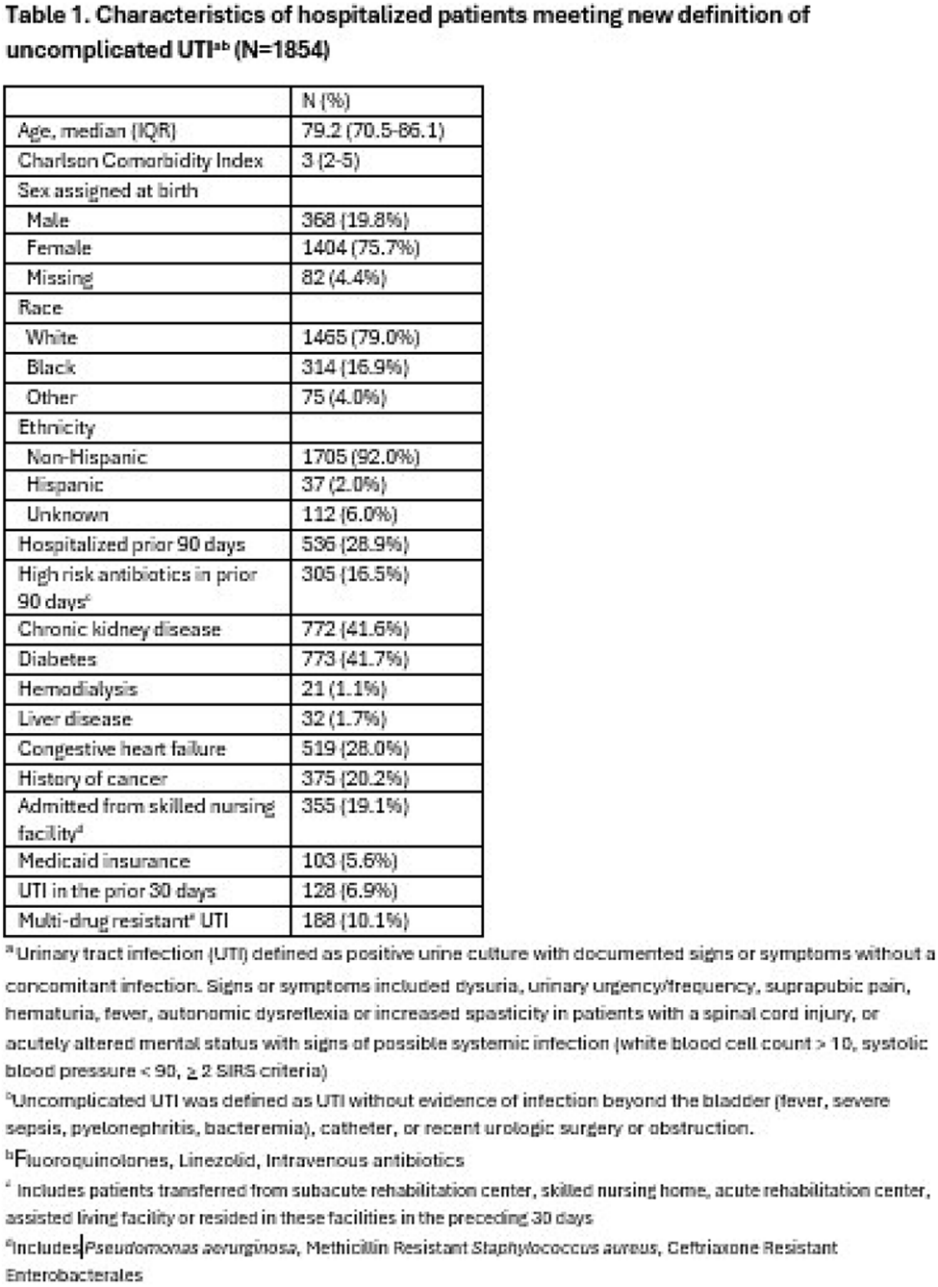

**Figure:**
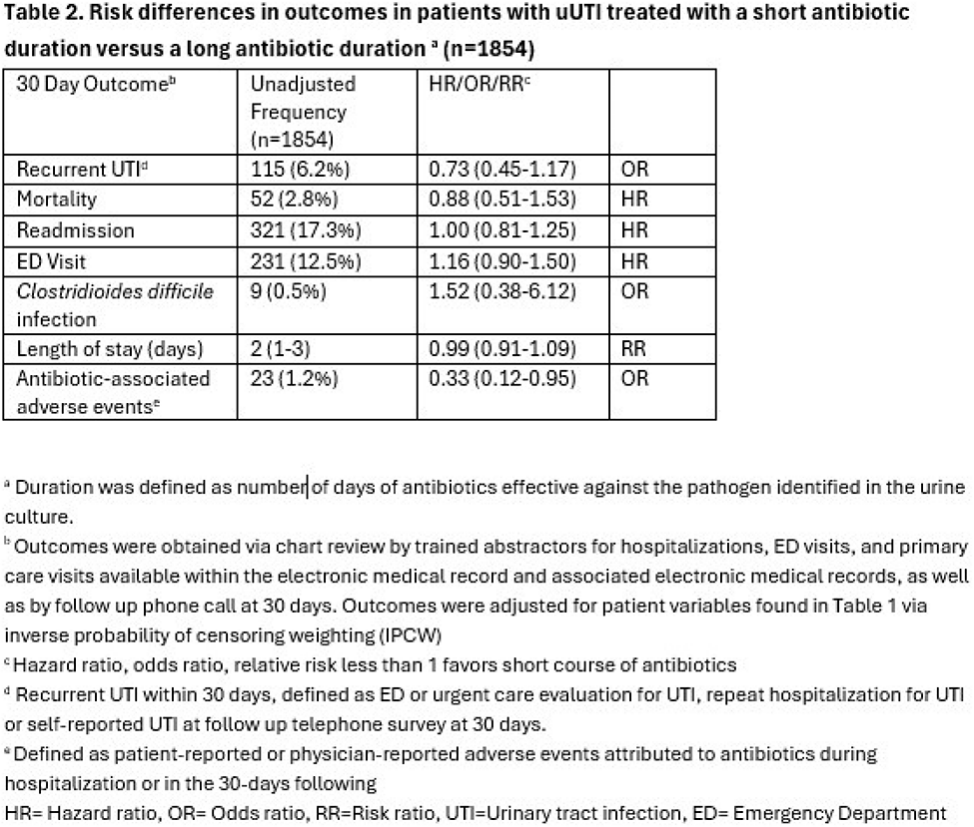

**Methods:**

From 11/2021 to 11/2024, data (e.g., patient characteristics, lab results, clinical outcomes) were collected in patients with a UTI at 68 hospitals in Michigan. We used a target trial emulation framework to assess treatment duration in patients meeting the new uUTI definition using a clone and censor approach. Short (3 - 5 days) vs long (6 - 14 days) antibiotic duration was compared for the primary outcome of 30-day recurrent UTI, as well as secondary clinical outcomes. Outcomes were adjusted via inverse probability of censor weights. Randomization occurred on treatment day 3. Patients were excluded if they received < 3 or > 14 days of effective antibiotics, or if they had signs/symptoms of infection beyond the bladder (e.g., fever, bacteremia, pyelonephritis), catheter-associated UTI, recent urologic surgery or obstruction.

**Results:**

Of 13,784 patients hospitalized with a UTI, 24.8% (N=1854) met the new uUTI definition and were eligible. 19.8% (N=368) were men [Table 1]. Under half (45.4%; n=841) were treated with a short antibiotic duration. Patients treated with short vs long duration had similar comorbidities, including Charlson Comorbidity Index. The most common empiric antibiotic used was ceftriaxone. After adjustment, there was no difference in 30-day recurrent UTI when comparing short vs long duration (OR: 0.73; 95% CI: 0.45-1.17) [Table 2]. A short antibiotic duration was associated with a decreased risk of 30-day antibiotic-related adverse events. (OR: 0.33; 95% CI: 0.12-0.95).

**Conclusion:**

Hospitalized patients meeting the new definition of uUTI treated with a short antibiotic duration had no difference in recurrent UTI compared to long duration, but short duration was associated with less antibiotic-associated adverse events. Our findings support the safety of shorter antibiotic durations for patients meeting the new definition of uUTI.

**Disclosures:**

All Authors: No reported disclosures

